# Effectiveness of Home-Based Progressive Resistance Exercise in the Management of Breast Cancer-Related Lymphedema: A Randomized Controlled Trial

**DOI:** 10.3390/jcm15187109

**Published:** 2026-09-13

**Authors:** Suzan Aydın, Sevinj Rafili, Ömer Şevgin

**Affiliations:** 1 Department of Physiotherapy and Rehabilitation, Faculty of Health Sciences, İstanbul Gelişim University, 34310 Istanbul, Türkiye; suaydin@gelisim.edu.tr; 2 Department of Physiotherapy and Rehabilitation, Faculty of Health Sciences, Üsküdar University, 34768 Istanbul, Türkiye; sevinj.rafili@st.uskudar.edu.tr

**Keywords:** quality of life, sleep quality, muscle strength, upper extremity, physical therapy modalities

## Abstract

**Background/Objectives:** Resistance exercise is considered safe in breast cancer-related lymphedema (BCRL), but its efficacy as an adjunct to standard decongestive care in women with established lymphedema is uncertain, and sleep quality is rarely assessed. This trial evaluated a home-based progressive resistance exercise programme in this population. **Methods:** Fifty women with unilateral upper-limb BCRL were randomly allocated (1:1) to exercise or control groups. Both groups received classic lymphedema physiotherapy twice weekly for 12 weeks; the exercise group additionally performed eight upper-limb exercises (three sets of ten repetitions, elastic resistance progressed every four weeks) twice weekly at home. The primary outcome was the percentage of excess limb volume derived from circumferential measurements. Assessors were blinded; groups were compared by analysis of covariance adjusted for baseline values. **Results:** Forty-six participants (92%) completed follow-up; adherence was 91.7% and no adverse events occurred. Excess limb volume decreased more in the exercise group (adjusted difference −2.04 percentage points, 95% CI −3.99 to −0.10; *p* = 0.040; *d* = 0.61), a finding robust in intention-to-treat and age-adjusted analyses. Handgrip strength and the LYMQOL function domain also favoured the exercise group, but no secondary outcome survived Holm–Bonferroni correction. Sleep quality improved comparably in both groups (*p* = 0.629). **Conclusions:** Home-based progressive resistance exercise added to lymphedema physiotherapy reduced excess limb volume, with a moderate effect and no adverse events, although the absolute between-group difference was small and its clinical importance uncertain. Sleep quality did not improve differentially and may require targeted intervention.

## 1. Introduction

Breast cancer-related lymphedema (BCRL) is among the most persistent and burdensome late effects of breast cancer treatment. It arises when axillary lymph node dissection, radiotherapy, or a combination of treatments disrupts lymphatic transport capacity, leading to the accumulation of protein-rich interstitial fluid in the affected upper limb [1]. The pooled incidence of unilateral arm lymphedema after breast cancer has been estimated at 16.6%, with a roughly fourfold higher risk after axillary lymph node dissection than after sentinel node biopsy [2]. Beyond the visible increase in limb volume, BCRL produces a cluster of impairments—reduced range of motion, muscle weakness, heaviness, pain, and recurrent infection risk—that restrict activities of daily living, increase cancer-related fatigue and diminish quality of life [1,3,4]. As survival after breast cancer continues to improve, the long-term management of this chronic condition has become a central concern of cancer rehabilitation.

Complete decongestive therapy, combining manual lymphatic drainage, compression, skin care and remedial exercise, remains the reference treatment for peripheral lymphedema [1,5]. Women with or at risk of BCRL were long advised to avoid loading the affected limb, but the Physical Activity and Lymphedema (PAL) trial showed that slowly progressive weight lifting in women with stable BCRL did not worsen limb volume and reduced both exacerbations and symptom severity [6]. Subsequent systematic reviews have converged on the same conclusion [7,8,9], and a recent dose–response meta-analysis reported that resistance training reduces lymphedema and improves upper-limb muscle strength, with larger effects at higher training doses [10].

The safety question is therefore largely settled. What remains unresolved is efficacy, and here the literature is more fragmented than it first appears, for three reasons.

First, the evidence base is weighted towards prevention rather than treatment. Trials delivering progressive resistance training from the day of surgery found no effect on arm volume or on the incidence of lymphedema [11], and meta-analyses of postoperative exercise-based rehabilitation report similarly limited effects on lymphedema outcomes [12]. These studies address a different clinical question from the one facing a clinician whose patient already has established lymphedema, where excess volume is present and therefore modifiable.

Second, the few trials conducted in women with established BCRL have been small and have produced inconsistent results for volume outcomes. Park et al. randomised twenty patients to progressive resistance exercise with elastic resistance or to a self-administered home programme, both alongside complete decongestive therapy, and found significant within-group reductions in edema volume but no between-group difference [13]; with sixteen completers, that trial was not powered to detect a between-group effect of plausible magnitude.

Third, the outcome sets used in this field have been narrow, and sleep quality is rarely assessed, despite evidence that women with BCRL report significantly poorer sleep than survivors without lymphedema and attribute this disturbance to the burden of lymphedema management—night-time bandaging and compression garments—rather than to the swelling per se [14,15]. Poorer sleep in this group has also been linked to a higher risk of obstructive sleep apnoea [16], and decongestive treatment has been reported to improve both fatigue and sleep quality [17]. Exercise improves sleep in other clinical populations, including obstructive sleep apnoea [18] and obesity [19], but whether the same holds in BCRL, where sleep disturbance may arise from the burden of treatment rather than from deconditioning, has not been tested.

The present trial was designed to address these gaps. In women with established upper-limb lymphedema following breast cancer treatment, we compared a twelve-week home-based progressive resistance exercise programme delivered in addition to classic lymphedema physiotherapy against classic lymphedema physiotherapy alone. The primary aim was to determine the effect of the programme on the percentage of excess limb volume in the affected limb. Secondary aims were to evaluate its effects on upper-limb function, muscle strength, fatigue, lymphedema-specific quality of life, and sleep quality. We hypothesised that the reduction in excess limb volume would be greater in the exercise group than in the control group, and that the exercise group would show greater improvement in each of the secondary outcomes.

## 2. Materials and Methods

### 2.1. Trial Design and Setting

This was a two-arm, parallel-group, single-centre randomized controlled superiority trial with 1:1 allocation, conducted at the Re-Heal Physiotherapy and Healthy Living Center, Adana, Türkiye. The trial is reported in accordance with the CONSORT 2010 statement [20]. Participants were recruited between March 2026 and April 2026, and follow-up was completed in August 2026.

### 2.2. Participants

Eligible participants were women aged 30 to 60 years who had developed unilateral upper-limb lymphedema following treatment for breast cancer (surgery, chemotherapy and/or radiotherapy), who were at least three months post-surgery, and who were able to tolerate compression bandaging or a compression garment. Lymphedema was diagnosed clinically by a physician. Clinical (ISL) stage was not recorded prospectively as a separate study variable; lymphedema severity was therefore characterised objectively using the percentage of excess limb volume derived from bilateral circumferential measurements, in accordance with the volume-based severity categories of the International Society of Lymphology, in which an excess volume of 5–20% denotes mild, 20–40% moderate and >40% severe lymphedema [1]. At screening, all referred patients were judged clinically by the assessing physician to have moderate lymphedema; the volume-based categories reported below were derived from the baseline circumferential measurements and are presented separately, since clinical judgement and volumetric classification do not necessarily coincide. The degree of tissue fibrosis and adipose deposition was likewise not formally graded at baseline (see Section 4.6). Participants were excluded if they required reinitiation of active cancer treatment during the study period, or if they were within the first three months after surgery. Women who had undergone lymphatic reconstructive surgery (lymphovenous anastomosis or vascularized lymph node transfer), whether preventive or therapeutic, were also ineligible; no participant had a history of lymphatic reconstruction.

### 2.3. Randomization and Allocation Concealment

After completion of the baseline assessment, participants were allocated in a 1:1 ratio using simple unrestricted computer-generated randomization; no blocking or stratification was applied. The allocation sequence was generated before the start of enrolment by an independent researcher with no role in recruitment, baseline assessment, intervention delivery, outcome assessment or statistical analysis.

Allocation was concealed using sequentially numbered, opaque, sealed envelopes prepared in accordance with the generated sequence. Envelopes were opened consecutively, and only after a participant had been formally enrolled and the baseline assessment completed. Participants were enrolled by an independent researcher and were assigned to groups by a second independent researcher; neither was involved in sequence generation, intervention delivery, outcome assessment or analysis. After completion of the trial, the allocation sequence and log were independently cross-checked by a researcher who had taken no part in recruitment, intervention delivery, outcome assessment or analysis.

### 2.4. Blinding

Owing to the nature of an exercise intervention, neither participants nor the treating physiotherapists could be blinded. The outcome assessor was blinded: all baseline and post-intervention measurements were performed by a physiotherapist unaware of group assignment who had no involvement in delivering either programme. Participants were instructed not to disclose their allocation to the assessor, assessments were conducted in a room separate from the treatment area, and treatment records were kept inaccessible to the assessor.

### 2.5. Interventions

#### 2.5.1. Standard Care (Both Groups)

All participants received classic lymphedema physiotherapy twice weekly for 12 weeks (24 sessions), delivered by a physiotherapist experienced in lymphedema management. Each session comprised manual lymphatic drainage (30 min), multilayer short-stretch compression bandaging applied at the end of the session, and simple lymphatic exercises (15 min). Standard compression bandaging was used in both groups throughout the study period. Structured skin-care education with written material was delivered at the start of the programme. Intermittent pneumatic compression was not used.

#### 2.5.2. Experimental Group

In addition to standard care, the exercise group performed a home-based progressive resistance exercise programme for the upper limb twice weekly on non-consecutive days for 12 weeks (24 prescribed sessions). All sessions were performed while wearing the compression bandaging already in place as part of standard care; no additional compression was applied for the purpose of the exercise programme.

Each session lasted approximately 40–45 min and comprised a 10 min warm-up (diaphragmatic breathing and active stretching of the cervical, pectoral and scapular regions), a 20–25 min resistance component, and a 10 min cool-down. The resistance component consisted of eight exercises performed bilaterally and alternately using Thera-Band elastic resistance bands (Performance Health, Warrenville, IL, USA): shoulder flexion, shoulder abduction, shoulder external rotation, elbow flexion, elbow extension, wrist flexion, wrist extension, and scapular retraction and protraction. Three sets of 10 repetitions were performed per exercise, with 60 s rest between sets.

Because the programme was home-based and elastic resistance does not permit load to be expressed as a percentage of the one-repetition maximum, intensity was prescribed by band colour and monitored using the Borg CR10 scale, with a target range of 4–6. Training commenced with the yellow band, and resistance was progressed by one band colour every four weeks (yellow, weeks 1–4; red, weeks 5–8; green, weeks 9–12), provided the participant could complete the final set of 10 repetitions at a perceived exertion of 4 or below; otherwise the participant remained at the current colour. Mean tension at 100% elongation is approximately 15.7% for yellow, 21.1% for red and 23.5% for green [21]. The programme was consistent with current exercise oncology recommendations for cancer survivors [22].

Before commencing, each participant attended one individual instruction session with a physiotherapist, during which every exercise was demonstrated and performed under supervision until correct technique was achieved. Participants received an illustrated exercise booklet and the corresponding Thera-Band set.

#### 2.5.3. Adherence and Safety Monitoring

Adherence was monitored by two methods. Participants recorded each completed session in a structured exercise log, collected at the final assessment. In addition, a team member not involved in outcome assessment telephoned each participant every 15 days throughout the intervention (maximum six calls) to review the log, reinforce technique, enquire about limb symptoms and address difficulties. Adherence was calculated as the proportion of the 24 prescribed sessions completed.

Participants were instructed to interrupt the programme and contact the research team if heaviness, tightness, pain or swelling of the affected limb increased. Where symptoms persisted beyond 24 h, resistance was to be reduced by one band colour and progression resumed only after resolution. An adverse event was defined a priori as an episode of cellulitis, an increase of ≥10% in excess limb volume, or worsening of limb symptoms requiring modification or discontinuation of the programme.

#### 2.5.4. Control Group

The control group received classic lymphedema physiotherapy alone and was not prescribed any resistance exercise programme during the study period.

### 2.6. Outcome Measures

All outcomes were assessed at baseline and immediately after the 12-week intervention. All patient-reported instruments were administered in Turkish, using versions for which cross-cultural adaptation and psychometric validation in Turkish-speaking populations have been published.

*Primary outcome—excess limb volume.* Limb circumference was measured with a flexible tape at 5 cm intervals from the ulnar styloid process to 40 cm proximally, in both limbs. Segmental volumes were calculated using the truncated cone formula and summed to give total limb volume [23]. The percentage of excess volume was calculated as the difference between affected and unaffected limb volumes, expressed as a percentage of the unaffected limb volume. Volume of the unaffected limb served as an internal control.

*Upper-limb function.* The Disabilities of the Arm, Shoulder and Hand questionnaire was used in the Turkish adaptation of Düger et al. (DASH-T), which demonstrated adequate test–retest reliability and construct validity in Turkish patients [24]. Scores range from 0 to 100; higher scores indicate greater disability.

*Muscle strength.* Handgrip strength of the affected limb was measured with a hydraulic hand dynamometer (Jamar; Performance Health, Warrenville, IL, USA) in the seated position using the standardised protocol of the American Society of Hand Therapists. Three trials were performed and the mean used for analysis.

*Fatigue.* Average fatigue over the preceding seven days was rated on an 11-point numeric rating scale (NRS) from 0 (no fatigue) to 10 (worst imaginable fatigue). As a single-item unidimensional rating scale, the NRS does not entail a separate psychometric validation procedure.

*Lymphedema-specific quality of life.* The Lymphedema Quality of Life questionnaire—Arm (LYMQOL-Arm) was used [25], in the Turkish version translated and validated by Bakar et al. in patients with BCRL [26]. It yields four domain scores (function, appearance, symptoms, mood), each 1–4 with higher scores indicating poorer quality of life, and a single overall quality-of-life item scored 1–10 with higher scores indicating better quality of life.

*Sleep quality.* The Pittsburgh Sleep Quality Index (PSQI) [27] was used in the Turkish version validated by Ağargün et al. [28]. The global score ranges from 0 to 21; higher scores indicate poorer sleep quality, and a score above 5 indicates poor sleep quality.

### 2.7. Sample Size

The sample size was determined a priori using G*Power (version 3.1.9.7; Heinrich Heine University Düsseldorf, Düsseldorf, Germany), based on a between-group effect size of Cohen’s *d* = 0.93 for objective lymphedema assessment reported in a pilot randomized controlled trial of resistance exercise in patients with BCRL [29]. With a two-sided α = 0.05, power (1 − β) of 0.80 and 1:1 allocation, a minimum of 20 participants per group (40 in total) was required. Allowing for approximately 20% attrition, the target was increased to 25 per group, giving a total of 50.

### 2.8. Statistical Analysis

Analyses were performed using IBM SPSS Statistics for Windows, version 26.0 (IBM Corp., Armonk, NY, USA). Distribution of continuous variables was examined using the Shapiro–Wilk test and visual inspection of histograms and Q–Q plots. Baseline characteristics were compared using the Mann–Whitney U test for continuous variables and the chi-square test for categorical variables.

Within-group changes were assessed using the Wilcoxon signed-rank test. The primary between-group comparison was performed by analysis of covariance, with the post-intervention value as dependent variable, group as fixed factor and the corresponding baseline value as covariate; this approach was preferred to comparison of change scores because it provides greater statistical efficiency and adjusts for residual baseline imbalance. Comparison of change scores using the Mann–Whitney U test was performed as a sensitivity analysis. The same model was applied to each secondary outcome.

Three prespecified sensitivity analyses were performed for the primary outcome: (i) an intention-to-treat analysis including all 50 randomised participants, in which missing post-intervention values were replaced by the corresponding baseline value (no-change imputation); (ii) an analysis of covariance additionally adjusted for age; and (iii) a responder analysis in which a participant was classified as a responder if the relative reduction in excess limb volume from baseline was 10% or greater, with groups compared using Fisher’s exact test. In addition, the change in volume of the unaffected limb was used to estimate the measurement error of the circumferential method, expressed on the scale of the primary outcome.

Standardised effect sizes are reported as Cohen’s *d* with 95% confidence intervals. To control the family-wise error rate across the five prespecified secondary outcomes, the Holm–Bonferroni procedure was applied. Participants without post-intervention data were excluded from the analysis of the affected outcomes; participants with a single missing questionnaire item were excluded from the analysis of that scale only. A two-sided *p* < 0.05 was considered statistically significant.

In accordance with CONSORT item 6b, we note that the trial registry initially listed the LYMQOL-Arm and the DASH as primary outcomes; the primary outcome of this report is the percentage of excess limb volume, as specified in the ethics-approved study protocol and used for the sample size calculation.

## 3. Results

### 3.1. Participant Flow and Baseline Characteristics

**Figure 1:**
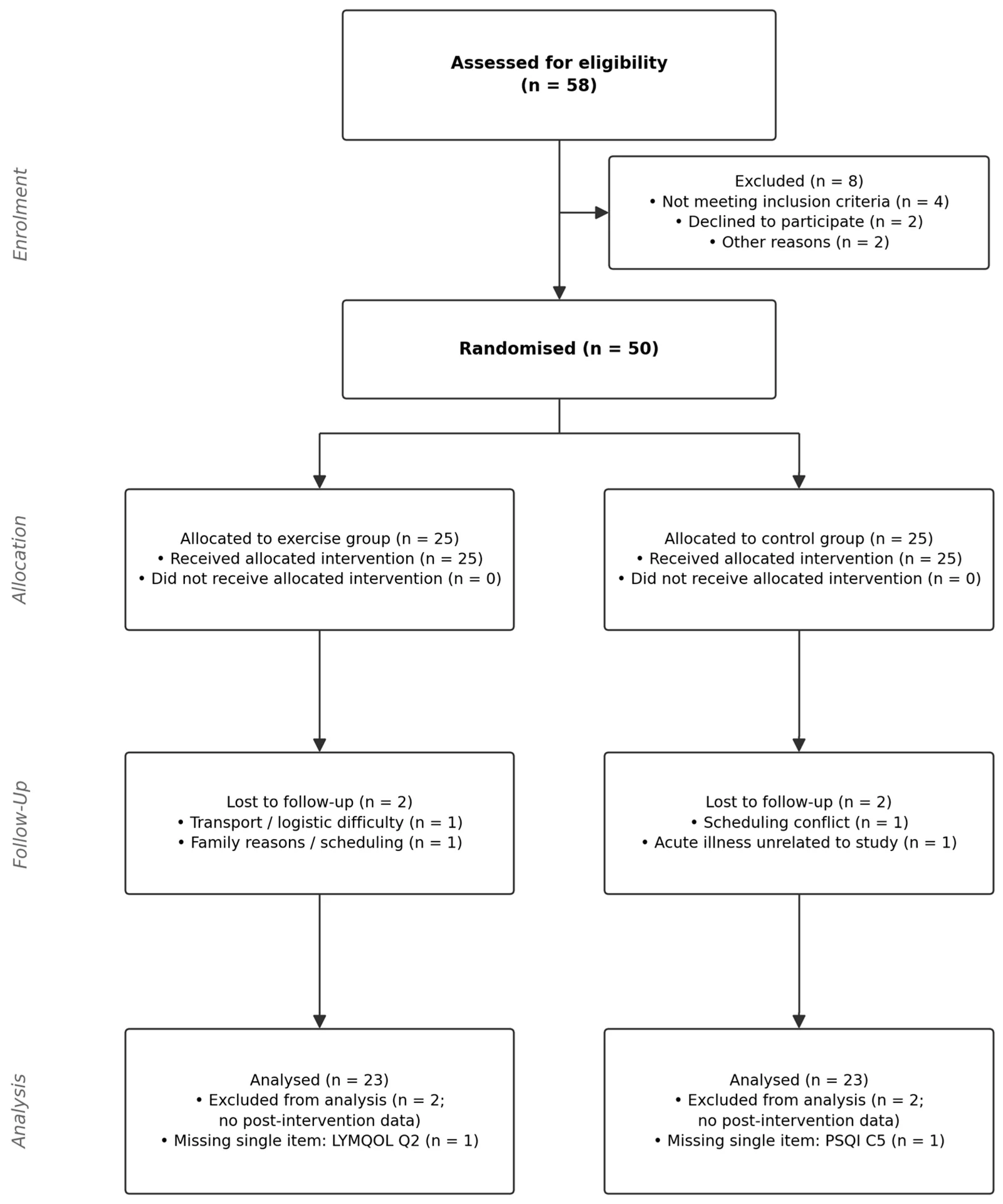
CONSORT flow diagram of participants through the trial.

Fifty women with BCRL were randomised, 25 to the exercise group and 25 to the control group (Figure 1). Four participants (8%) were lost to follow-up—two in each group—leaving 23 per group with complete post-intervention data. Reasons were transport or logistic difficulty (*n* = 1), family reasons and scheduling conflict (*n* = 2), and an acute illness unrelated to the study (*n* = 1). Two participants who completed follow-up had a single missing questionnaire item (LYMQOL item 2, *n* = 1; PSQI component 5, *n* = 1) and were excluded from the analysis of the affected scale only.

Baseline demographic and clinical characteristics are presented in Table 1. Individual participant-level baseline data for all 50 randomised participants are provided in Supplementary Table S1. The groups were comparable on all measured characteristics (all *p* > 0.05). Adherence to the home-based programme, calculated from the exercise logs, was high: participants completed a mean of 22 of the 24 prescribed sessions (91.7%). All scheduled telephone contacts were completed, giving six contacts per participant over the 12-week intervention period.

Baseline excess limb volume ranged from 11.9% to 45.8% across the sample. Applying the volume-based severity criteria of the International Society of Lymphology, 14 participants (28%) fell into the mild category (5–20% excess volume), 34 (68%) into the moderate category (20–40%) and 2 (4%) into the severe category (>40%), with a comparable distribution in the two groups. The sample therefore spanned a range of lymphedema severity rather than being confined to a single category.

### 3.2. Primary Outcome: Excess Limb Volume

Excess limb volume decreased in both groups over the 12-week period, from 23.22 ± 8.79% to 19.60 ± 9.16% in the exercise group (mean change −3.62 ± 3.09%, *p* < 0.001) and from 26.14 ± 7.53% to 24.47 ± 7.92% in the control group (mean change −1.66 ± 3.29%, *p* = 0.045).

**Figure 2:**
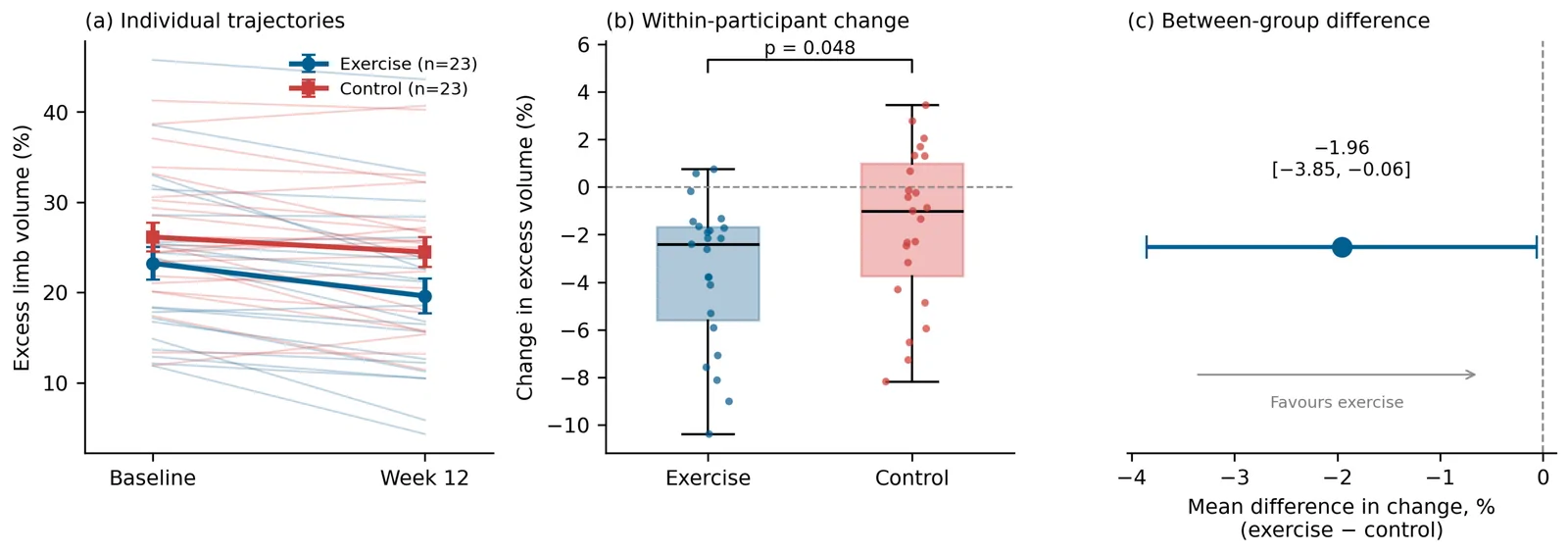
Primary outcome. (**a**) Individual participant trajectories for excess limb volume from baseline to week 12; thick lines represent group means ± standard error. (**b**) Distribution of within-participant change; boxes show median and interquartile range, whiskers extend to 1.5 × IQR, individual data points overlaid. (**c**) Unadjusted mean between-group difference in change scores with 95% confidence interval (−1.96 percentage points, 95% CI −3.85 to −0.06; sensitivity analysis, *p* = 0.048). The primary covariate-adjusted estimate (−2.04 percentage points, 95% CI −3.99 to −0.10) is reported in the text and Table 2.

The reduction was significantly greater in the exercise group. Analysis of covariance adjusting for baseline values showed an adjusted between-group difference of −2.04 percentage points (95% CI −3.99 to −0.10; *p* = 0.040), corresponding to *d* = 0.61 (95% CI 0.02 to 1.21). The sensitivity analysis comparing change scores gave a consistent result (*p* = 0.048). Individual trajectories, the distribution of within-participant change and the between-group difference are shown in Figure 2.

The finding was robust across all three sensitivity analyses. In the intention-to-treat analysis including all 50 randomised participants, the adjusted between-group difference was −1.86 percentage points (95% CI −3.69 to −0.04; *p* = 0.046). After additional adjustment for age, it was −1.98 percentage points (95% CI −3.95 to −0.00; *p* = 0.050). The mean relative reduction in excess limb volume was 17.8 ± 17.4% in the exercise group and 6.1 ± 14.9% in the control group, and 15 of 23 participants (65%) in the exercise group achieved a relative reduction of at least 10%, compared with 8 of 23 (35%) in the control group (risk difference 30.4%, 95% CI 2.9 to 58.0; odds ratio 3.52; Fisher’s exact *p* = 0.076; number needed to treat 3.3).

To place the observed effect in the context of measurement precision, the change in volume of the unaffected limb was used as an estimate of measurement error, since this limb was not targeted by the intervention. The standard deviation of this change was 16.6 mL against a mean unaffected limb volume of 2282 mL, corresponding to approximately 0.73 percentage points on the excess volume scale. The observed between-group difference of 2.04 percentage points (≈47 mL) therefore exceeded the estimated measurement error by a factor of approximately 2.8.

### 3.3. Secondary Outcomes

**Figure 3:**
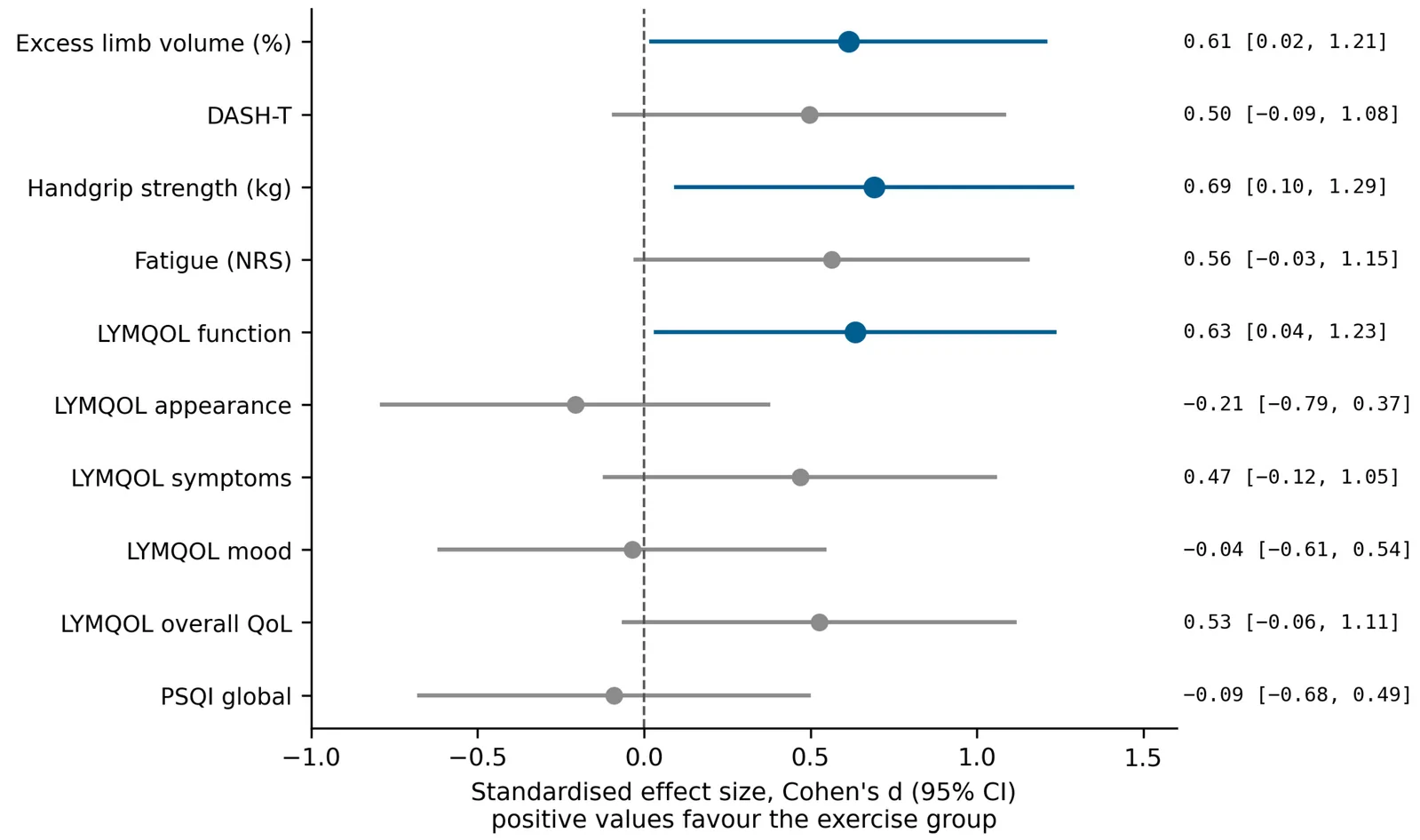
Between-group standardised effect sizes (Cohen’s *d*) with 95% confidence intervals for all outcomes. Positive values favour the exercise group. Outcomes whose confidence interval excludes zero are shown in blue. The dashed vertical line indicates no between-group difference (*d* = 0).

Results for all secondary outcomes are presented in Table 2 and summarised in Figure 3.

*Muscle strength.* Handgrip strength increased in both groups, with a significantly greater increase in the exercise group (adjusted difference +1.03 kg, 95% CI 0.13 to 1.93; *p* = 0.026; *d* = 0.69, 95% CI 0.10 to 1.29).

*Upper-limb function.* DASH-T scores improved in both groups, with a between-group difference favouring the exercise group that did not reach statistical significance (adjusted difference −2.37 points, 95% CI −5.01 to 0.28; *p* = 0.078; *d* = 0.50, 95% CI −0.09 to 1.08).

*Lymphedema-specific quality of life.* Of the five LYMQOL domains, only the function domain showed a significant between-group difference (adjusted difference −0.25, 95% CI −0.49 to −0.01; *p* = 0.042; *d* = 0.63, 95% CI 0.04 to 1.23). Differences in the symptoms, appearance, mood and overall quality-of-life domains were not statistically significant.

*Fatigue.* Fatigue decreased in both groups, with no significant between-group difference after adjustment (adjusted difference −0.32 points, 95% CI −0.80 to 0.15; *p* = 0.173).

*Sleep quality.* PSQI global scores improved to a similar extent in both groups (exercise −1.09 ± 1.16; control −1.18 ± 0.91), with no between-group difference (adjusted difference +0.16, 95% CI −0.51 to 0.83; *p* = 0.629; *d* = −0.09, 95% CI −0.68 to 0.49).

*Correction for multiple comparisons.* Holm–Bonferroni correction was applied across the five prespecified secondary outcomes (handgrip strength, DASH-T, fatigue, PSQI global score and LYMQOL overall quality of life). After correction, none of the between-group differences remained statistically significant (handgrip strength, adjusted *p* = 0.130; DASH-T, adjusted *p* = 0.312; LYMQOL overall quality of life, adjusted *p* = 0.312; fatigue, adjusted *p* = 0.346; PSQI, adjusted *p* = 0.629). The four LYMQOL domain scores were analysed as exploratory outcomes; when the correction is extended to all nine secondary comparisons in Table 2, no difference remains statistically significant, including the LYMQOL function domain (adjusted *p* = 0.336). The secondary findings should therefore be regarded as exploratory and hypothesis-generating.

### 3.4. Safety

No adverse events were recorded in either group during the 12-week intervention period. No episode of cellulitis occurred, no participant reported a symptomatic exacerbation of lymphedema requiring modification or discontinuation of the exercise programme, and no participant discontinued the intervention because of limb symptoms.

Volume of the unaffected limb, measured as an internal control, did not change significantly in either group (exercise −2.3 ± 15.9 mL, *p* = 0.345; control −3.5 ± 17.6 mL, *p* = 0.445), indicating that the programme did not induce edema in the contralateral limb.

## 4. Discussion

In this randomized controlled trial, in which forty-six of the fifty randomised women with established BCRL completed follow-up and were analysed, adding a twelve-week home-based progressive resistance exercise programme to classic lymphedema physiotherapy produced a greater reduction in excess limb volume than lymphedema physiotherapy alone. The adjusted between-group difference was 2.04 percentage points (95% CI −3.99 to −0.10; *p* = 0.040), corresponding to a moderate standardised effect (*d* = 0.61). Handgrip strength also improved more in the exercise group, and the LYMQOL function domain showed a between-group difference in the adjusted analysis; neither survived correction for multiple comparisons. Sleep quality improved similarly in both groups. No adverse events occurred, and volume of the unaffected limb was unchanged.

### 4.1. Volume Outcomes in the Context of Previous Trials

Our primary finding both extends and clarifies the existing evidence. The most directly comparable trial is that of Park et al., who also delivered elastic-resistance progressive exercise alongside complete decongestive therapy in women with established BCRL [13]. They observed significant within-group reductions in edema volume in both arms but no significant difference between them. Our study reproduces their within-group pattern but, unlike theirs, detects a between-group difference.

Three design differences plausibly account for this divergence. The most important is statistical power: sixteen participants completed the Park trial, against forty-six here. A between-group effect of the magnitude we observed would have been undetectable in a sample of that size. The second is intervention duration: their programme ran for six weeks, ours for twelve, and if the effect of resistance training on interstitial fluid dynamics accumulates gradually, a six-week exposure may be too brief. The third is the equalisation of background care: both of our groups received an identical dose of classic lymphedema physiotherapy, so that the between-group contrast isolates the incremental contribution of resistance exercise rather than confounding it with differences in decongestive treatment.

A further point concerns baseline balance in the primary outcome. Despite randomization, mean baseline excess limb volume was somewhat higher in the control group than in the exercise group (26.1% versus 23.2%), a difference compatible with chance under simple unrestricted randomization but consistent with slightly more advanced lymphedema in the control arm. Two features of the analysis mitigate the risk that this imbalance generated the observed effect. First, the primary analysis was an analysis of covariance with the baseline value as the covariate, which adjusts the between-group comparison for baseline differences. Second, both regression to the mean and the greater absolute scope for volume reduction in a larger limb would, if anything, be expected to favour the control group, so any residual bias from this imbalance is more plausibly conservative than inflationary. The imbalance is nevertheless acknowledged as a limitation (Section 4.6).

Our findings should be distinguished from the prevention literature. Ammitzbøll et al. found no effect of progressive resistance training on arm volume when the intervention was initiated immediately after surgery in women who did not yet have lymphedema [11]. That null result and our positive one are not in conflict; they answer different questions. In a limb with no excess volume there is nothing for the intervention to reduce, whereas in the population studied here—women with a mean excess volume of approximately 25% at baseline—there is substantial scope for change. Framing resistance training as a treatment adjunct rather than a preventive measure appears, on this evidence, to be the more defensible clinical position [12].

The home-based format also merits comment. Home exercise programmes have recently been shown to reduce lymphedema incidence and support its management in breast cancer populations [30], and the present findings extend that evidence to the treatment of established lymphedema. Because the programme required only elastic resistance bands and a single instruction session, it is readily transferable to settings in which supervised facility-based training is not available.

Our results are also consistent with the safety conclusions of the PAL trial and subsequent systematic reviews [6,7,8,9]. We recorded no adverse events, no episodes of cellulitis and no symptomatic exacerbations. The unaffected limb, serving as an internal control, showed no significant volume change in either group. Together with the observed volume reduction in the affected limb, these data support the position that appropriately progressed resistance exercise, performed under compression, is safe in this population.

### 4.2. Training Dose

We powered this trial on an effect size of *d* = 0.93, derived from a pilot randomized trial of forearm resistance exercise in BCRL [29]. The effect observed was smaller (*d* = 0.61). Two considerations bear on this discrepancy.

The first is methodological: effect sizes derived from small pilot trials are systematically inflated, and a pilot with eighteen participants provides an imprecise basis for sample size estimation. A downward correction on moving to a larger sample is an expected outcome.

The second is specific and more informative. Our programme delivered two sessions per week. The recent dose–response meta-analysis in this field found that larger effects on lymphedema were associated with higher training intensity (5–8 repetition maximum), four sessions per week and a weekly volume of 120–180 min [10]. A separate meta-analysis applying the ACSM FITT framework reached a compatible conclusion, with greater benefit at higher adherence to the recommended exercise dose [31]. Our protocol sat below that threshold on both frequency and weekly volume. Critically, adherence exceeded 90%, so the shortfall cannot be attributed to poor compliance: participants completed very nearly all of what was prescribed, and what was prescribed was, by current dose–response evidence, a conservative dose. This suggests that the moderate effect observed here represents a floor rather than a ceiling, and that trials testing higher-frequency or higher-intensity protocols are warranted.

### 4.3. Muscle Strength and Upper-Limb Function

Handgrip strength increased more in the exercise group (adjusted difference +1.03 kg; *d* = 0.69), despite the programme containing no dedicated gripping exercise. Two mechanisms are plausible. First, elastic-resistance training inherently loads the grip: the participant must hold the band throughout every repetition, so grip demand rises as band resistance rises—an explanation advanced by Park et al. for the same finding [13]. Second, and specific to our protocol, wrist flexion and extension exercises provided direct forearm loading that comparator protocols did not contain. There may also be a lymphatic dimension: forearm blood flow peaks at approximately 30% of maximal grip force, and the accompanying increase in capillary permeability has been proposed to enhance lymph formation [32], linking grip work to fluid dynamics rather than to strength alone.

DASH-T scores improved in both groups, with a between-group difference favouring the exercise group that did not reach conventional significance (*p* = 0.078; *d* = 0.50). Given the trial’s realised power, this is more plausibly read as inconclusive than as evidence of absence.

### 4.4. Sleep Quality

Sleep quality improved to a similar degree in both groups, with no between-group difference (*p* = 0.629; *d* = −0.09). We regard this as one of the more informative results of the trial rather than a simple failure to reject the null hypothesis, for two reasons.

First, it is an uncommon measurement in the literature. Despite consistent evidence that women with BCRL sleep worse than survivors without lymphedema [14,15,16], sleep quality is rarely included as an outcome in exercise trials in this population, and calls to integrate it into lymphedema outcome assessment have gone largely unanswered [15].

The absence of a differential effect is also notable against the wider exercise literature. Structured exercise improves sleep quality in other clinical populations, including patients with obstructive sleep apnoea [18] and individuals with obesity, in whom resistance training combined with dietary intervention improved both sleep and fatigue [19]. That a comparable resistance stimulus did not produce an additional sleep benefit here argues against a purely deconditioning-driven mechanism and supports a condition-specific explanation.

Second, the pattern of our data is consistent with a specific mechanistic account. Qualitative work indicates that sleep disturbance in BCRL is attributed by patients less to the swelling itself than to the apparatus of its management—night-time bandaging, compression garments, and the discomfort these entail [15]. If that account is correct, an intervention that reduces limb volume without altering the management burden would not be expected to improve sleep specifically, which is what we observed: excess volume fell significantly more in the exercise group, yet sleep improved identically in both. The parallel improvement is itself consistent with an effect of the decongestive care that both groups received, or with regression to the mean and non-specific effects of trial participation.

The clinical implication is that sleep disturbance in BCRL may require targeted intervention in its own right—addressing bandaging comfort, garment tolerability and sleep behaviour—rather than being expected to resolve as a downstream consequence of successful volume reduction. This hypothesis merits direct testing.

### 4.5. Clinical Relevance and the Context of Reconstructive Lymphatic Surgery

The statistical significance of the primary outcome should not be conflated with clinical importance. The adjusted between-group difference of 2.04 percentage points reduced mean excess limb volume from approximately 23% to 20% in the exercise group, so that women in both arms remained with substantial residual lymphedema, and no minimal clinically important difference has been established for excess limb volume in this population. Moreover, after correction for multiple testing no secondary outcome—including lymphedema-specific quality of life—differed significantly between groups, so the additional volume reduction was not accompanied by a demonstrable differential improvement in how participants felt or functioned within the 12-week horizon of the trial. The most defensible interpretation of our data is therefore that home-based resistance exercise produces a modest additional volume reduction whose clinical value, taken in isolation, remains uncertain. The argument for offering the programme rests less on the size of the average effect than on its favourable burden–benefit profile: it is inexpensive, home-based and safe, adherence exceeded 90%, and almost twice as many exercising participants achieved a relative volume reduction of at least 10% (65% versus 35%; number needed to treat 3.3). Whether these advantages justify a twelve-week commitment for an average additional reduction of approximately two percentage points is a decision to be shared with individual patients rather than presumed.

These findings should also be placed in the context of reconstructive lymphatic surgery. Microsurgical procedures—lymphaticovenous anastomosis (LVA) and vascularized lymph node transfer (VLNT)—achieve improvements of a different order of magnitude from conservative management: a systematic review and meta-analysis of 52 studies reported a pooled clinical improvement of approximately 36% in upper-extremity lymphedema, with 29% after LVA and 42% after VLNT [33], and a retrospective cohort of 112 patients with chronic BCRL documented sustained reductions over 24 months with both techniques, LVA producing an earlier response and VLNT a delayed but more durable one [34]. Neither the programme evaluated here nor decongestive therapy alone approaches effects of this size. For women with established BCRL whose response to optimised conservative treatment is inadequate—particularly in the absence of extensive fibroadipose change—referral to plastic surgery for assessment of suitability for lymphatic reconstruction should therefore form part of the treatment algorithm. The two approaches are, moreover, not competitors: a combination of lymphatic reconstruction with structured resistance training—for example as prehabilitation before, or adjunct therapy after, surgery—is biologically plausible, may act on complementary mechanisms, and merits prospective evaluation.

### 4.6. Strengths and Limitations

The principal strengths of this trial are its prospective registration, assessor-blinded design, equalisation of background decongestive care between groups, high retention (92%) and adherence (91.7%), the use of an internal control limb to verify safety, and the inclusion of sleep quality among the outcomes.

Several limitations should be considered. Regarding *statistical power*, the trial was powered on an anticipated effect size of *d* = 0.93; the realised effect was *d* = 0.61, for which achieved power was approximately 53%. The primary result is statistically significant but marginal, with the upper bound of the confidence interval close to zero, and the trial was underpowered for the secondary outcomes. No secondary between-group difference survived Holm–Bonferroni correction, and these should be regarded as exploratory.

Regarding *volume measurement*, excess limb volume was derived from circumferential measurements using the truncated cone formula. This method is reliable and clinically practical, and its validity against water displacement and optoelectronic volumetry is established [23], and it remains among the most widely used non-invasive assessment tools in this field [9]; however, it is less sensitive to changes in extracellular fluid than bioimpedance spectroscopy and less precise than perometry. It also cannot distinguish fluid from tissue composition changes, a relevant consideration in a resistance training trial where lean mass may increase. Relatedly, the tissue quality of the lymphedema—the degree of dermal fibrosis and fatty tissue deposition—was not formally assessed or graded at baseline. Because fibrotic, fat-dominant lymphedema responds less readily to volume-directed treatment than fluid-dominant swelling, the absence of tissue-composition data limits both the characterisation of the sample and the identification of the participants most likely to benefit; future trials should incorporate pitting assessment, tissue dielectric constant measurement, bioimpedance spectroscopy or imaging for this purpose.

Allocation was by simple unrestricted randomization, and the resulting exactly equal group sizes (25 per arm) arose by chance rather than by restriction of the allocation sequence. Simple randomization also permitted a modest baseline imbalance in the primary outcome: mean baseline excess limb volume was approximately three percentage points higher in the control group, suggesting somewhat more advanced lymphedema in that arm. Although the primary analysis adjusted for baseline values and the likely direction of any residual bias is conservative, such an imbalance would have been prevented by stratified or minimisation-based allocation, and it should be borne in mind when interpreting the results.

A further consideration concerns *unequal attention and contact between groups*. In addition to the exercise programme itself, participants in the exercise group received an individual instruction session, an illustrated exercise booklet, an exercise log, and telephone contact every 15 days, whereas the control group received none of these. Part of the observed between-group difference may therefore reflect the additional professional contact, behavioural monitoring and self-monitoring rather than the resistance exercise itself. Attention-controlled designs are needed to separate these components.

Compression exposure, by contrast, was comparable between groups: standard compression bandaging was used in both arms throughout the study period, and exercise sessions were performed while wearing the bandaging already in place rather than with additional compression applied for that purpose. The observed between-group difference is therefore unlikely to be attributable to differential compression, although the approximately 18 h during which the exercise group was both bandaged and physically active over the 12-week period cannot be entirely separated from the effect of the exercise itself.

A further limitation concerns outcome measurement and blinding of participants. The DASH-T, LYMQOL-Arm, PSQI and fatigue NRS are all patient-reported instruments, and participants were necessarily aware of their group allocation; handgrip dynamometry is also effort-dependent and therefore susceptible to participant expectation even when the assessor is blinded. Of the outcomes assessed, only limb volume derived from circumferential measurement is relatively protected from this source of bias, which further constrains interpretation of the secondary findings.

Regarding *adherence measurement*, adherence was calculated from participant-completed exercise logs and was not objectively verified; self-reported adherence is known to overestimate actual participation. Regarding *duration of follow-up*, outcomes were assessed immediately after the intervention, and whether the volume reduction is maintained after supervised contact and telephone follow-up cease is unknown. Regarding *blinding*, participants and treating physiotherapists could not be blinded, and the success of assessor blinding was not formally assessed. Regarding *interpretability*, no minimal clinically important difference has been established for the LYMQOL-Arm, and the clinical meaning of a 2-percentage-point between-group difference in excess limb volume requires cautious interpretation.

Finally, regarding *generalisability*, this was a single-centre trial in women at least three months post-surgery and able to tolerate compression. The findings may not extend to men with BCRL, to women with more severe or longer-standing lymphedema, or to settings where regular decongestive physiotherapy is unavailable.

## 5. Conclusions

In women with established breast cancer-related lymphedema, a twelve-week home-based progressive resistance exercise programme added to classic lymphedema physiotherapy produced a greater reduction in excess limb volume than lymphedema physiotherapy alone, with a moderate effect size and no adverse events. The absolute between-group difference was nevertheless modest—approximately two percentage points—and, in the absence of an established minimal clinically important difference and of a demonstrable quality-of-life benefit, its clinical importance requires cautious interpretation. Improvements in handgrip strength and lymphedema-specific functional quality of life were observed but did not survive correction for multiple testing. Sleep quality improved equally in both groups, suggesting that the sleep disturbance associated with lymphedema is not resolved by volume reduction alone and may require targeted intervention. Given that adherence was high while the prescribed dose was conservative by current dose–response standards, adequately powered trials of higher-frequency or higher-intensity resistance protocols, with longer follow-up, are the logical next step.

## Figures and Tables

**Table 1:** Baseline demographic and clinical characteristics.

Characteristic	Exercise Group (*n* = 25)	Control Group (*n* = 25)	*p*
Age, years	47.64 ± 7.44	48.60 ± 7.02	0.641
Height, cm	162.24 ± 8.05	162.20 ± 6.55	0.930
Body mass, kg	69.96 ± 11.32	72.76 ± 10.28	0.197
Body mass index, kg/m^2^	26.98 ± 6.33	27.78 ± 4.51	0.299
Number of children	1.76 ± 1.01	1.72 ± 1.06	>0.99
Time since surgery, months	24.00 ± 12.66	24.52 ± 11.78	0.756
Affected limb—right, *n* (%)	14 (56.0)	11 (44.0)	0.572
Currently employed, *n* (%)	14 (56.0)	13 (52.0)	>0.99
Education—university or above, *n* (%)	8 (32.0)	11 (44.0)	0.201
Income—middle, *n* (%)	14 (56.0)	12 (48.0)	0.803
Married, *n* (%)	17 (68.0)	16 (64.0)	0.879
Any comorbidity, *n* (%)	15 (60.0)	17 (68.0)	0.712
Regular medication use, *n* (%)	13 (52.0)	16 (64.0)	0.567
History of psychological disorder, *n* (%)	3 (12.0)	4 (16.0)	>0.99
Poor sleep quality at baseline (PSQI > 5), *n* (%)	16 (64.0)	18 (72.0)	0.762

Values are mean ± standard deviation unless otherwise stated (standard deviations are sample standard deviations, calculated with *n* − 1 in the denominator). Continuous variables were compared using the Mann–Whitney U test and categorical variables using the chi-square test. For education, income, marital status and comorbidity, *p* values refer to the comparison of the full category distributions listed in Supplementary Table S1. PSQI, Pittsburgh Sleep Quality Index.

**Table 2:** Primary and secondary outcomes at baseline and week 12.

Outcome	Exercise: Baseline	Exercise: Week 12	Exercise: Change	Control: Baseline	Control: Week 12	Control: Change	Adjusted Difference (95% CI) ^1^	*p* ^1^
Excess limb volume, %	23.22 ± 8.79	19.60 ± 9.16	−3.62 ± 3.09	26.14 ± 7.53	24.47 ± 7.92	−1.66 ± 3.29	−2.04 (−3.99 to −0.10)	**0.040**
Handgrip strength, kg	21.41 ± 2.43	23.21 ± 3.18	+1.80 ± 1.76	21.28 ± 2.54	22.04 ± 2.93	+0.76 ± 1.20	+1.03 (0.13 to 1.93)	**0.026**
DASH-T	52.33 ± 9.31	47.19 ± 9.29	−5.14 ± 4.67	56.04 ± 8.54	53.05 ± 9.59	−2.99 ± 3.96	−2.37 (−5.01 to 0.28)	0.078
Fatigue, NRS	4.61 ± 0.89	3.74 ± 1.45	−0.87 ± 0.92	5.30 ± 1.36	4.87 ± 1.60	−0.43 ± 0.59	−0.32 (−0.80 to 0.15)	0.173
PSQI global	6.78 ± 1.24	5.70 ± 1.69	−1.09 ± 1.16	7.64 ± 1.47	6.45 ± 1.90	−1.18 ± 0.91	+0.16 (−0.51 to 0.83)	0.629
LYMQOL function	2.67 ± 0.38	2.40 ± 0.55	−0.28 ± 0.34	2.85 ± 0.34	2.82 ± 0.49	−0.03 ± 0.43	−0.25 (−0.49 to −0.01)	**0.042**
LYMQOL appearance	2.31 ± 0.55	2.21 ± 0.58	−0.10 ± 0.34	2.33 ± 0.51	2.15 ± 0.59	−0.18 ± 0.40	+0.07 (−0.14 to 0.29)	0.496
LYMQOL symptoms	2.68 ± 0.49	2.26 ± 0.47	−0.42 ± 0.33	2.66 ± 0.41	2.45 ± 0.66	−0.21 ± 0.53	−0.21 (−0.47 to 0.06)	0.123
LYMQOL mood	2.13 ± 0.33	1.90 ± 0.40	−0.23 ± 0.34	2.26 ± 0.41	2.02 ± 0.60	−0.24 ± 0.32	+0.02 (−0.18 to 0.22)	0.844
LYMQOL overall QoL	5.97 ± 0.95	6.63 ± 1.20	+0.66 ± 0.96	5.77 ± 0.80	5.88 ± 1.45	+0.10 ± 1.13	+0.56 (−0.07 to 1.20)	0.080

Values are mean ± standard deviation; *n* = 23 per group unless otherwise stated (LYMQOL function, *n* = 22 exercise; PSQI, *n* = 22 control). ^1^ Analysis of covariance with the baseline value as covariate. A negative adjusted difference favours the exercise group for excess limb volume, DASH-T, fatigue, PSQI and the LYMQOL function, appearance, symptoms and mood domains; a positive difference favours the exercise group for handgrip strength and LYMQOL overall quality of life. DASH-T, Turkish version of the Disabilities of the Arm, Shoulder and Hand questionnaire; NRS, numeric rating scale; PSQI, Pittsburgh Sleep Quality Index; LYMQOL, Lymphedema Quality of Life questionnaire; CI, confidence interval. Values in bold indicate *p* < 0.05 before correction for multiple comparisons.

## Data Availability

The original contributions presented in this study are included in the article/Supplementary Materials. Further inquiries can be directed to the corresponding author.

## Supplementary Materials

The following supporting information can be downloaded at https://www.mdpi.com/article/10.3390/jcm15187109/s1: Table S1: Individual baseline demographic and clinical characteristics of the study participants (*n* = 50).

## References

[1] Executive Committee of the International Society of Lymphology (2023). The Diagnosis and Treatment of Peripheral Lymphedema: 2023 Consensus Document of the International Society of Lymphology. Lymphology.

[2] DiSipio T., Rye S., Newman B., Hayes S. (2013). Incidence of unilateral arm lymphoedema after breast cancer: A systematic review and meta-analysis. Lancet Oncol..

[3] Soltanipur M., Yarmohammadi H., Shahshenas S., Sheikhi Z. (2024). The relationship between upper-limb lymphedema and fatigue among breast cancer survivors. Eur. J. Cancer Care.

[4] Acikgoz Orhan S., Ozsaker E. (2025). Lymphedema, fatigue, and quality of life in breast cancer survivors with axillary lymph node dissection. Lymphat. Res. Biol..

[5] Leon-Diaz R., Medina-Otero A. (2025). Prevention and treatment of postmastectomy lymphedema: A physiotherapy perspective. Curr. Oncol..

[6] Schmitz K.H., Ahmed R.L., Troxel A., Cheville A., Smith R., Lewis-Grant L., Bryan C.J., Williams-Smith C.T., Greene Q.P. (2009). Weight lifting in women with breast-cancer-related lymphedema. N. Engl. J. Med..

[7] Hasenoehrl T., Palma S., Ramazanova D., Kölbl H., Dorner T.E., Keilani M., Crevenna R. (2020). Resistance exercise and breast cancer–related lymphedema—A systematic review update and meta-analysis. Support. Care Cancer.

[8] Shamsesfandabadi P., Shams Esfand Abadi M., Yin Y., Carpenter D.J., Peluso C., Hilton C., Coopey S.B., Gomez J., Beriwal S., Champ C.E. (2025). Resistance training and lymphedema in breast cancer survivors. JAMA Netw. Open.

[9] Arias-Crespo M., García-Fernández R., Calvo-Ayuso N., Liébana-Presa C., Martín-Vázquez C., Fernández-Martínez E. (2025). Impact of physical exercise on breast cancer-related lymphedema and non-invasive measurement tools: A systematic review. Cancers.

[10] Wang L., Liu Y., Zhang W., Wang Y., Zhai Z., Cheng H., Yao N. (2025). Effects of resistance training on breast cancer–related arm lymphedema: A systematic review and dose–response meta-analysis. Support. Care Cancer.

[11] Ammitzbøll G., Johansen C., Lanng C., Andersen E.W., Kroman N., Zerahn B., Hyldegaard O., Wittenkamp M.C., Dalton S.O. (2019). Progressive resistance training to prevent arm lymphedema in the first year after breast cancer surgery: Results of a randomized controlled trial. Cancer.

[12] Xue T., Zhang L., Zhang D. (2025). Exercise-based interventions for postoperative rehabilitation in breast cancer patients: A systematic review and meta-analysis of randomized controlled trials. Medicine.

[13] Park Y.-J., Na S.-J., Kim M.-K. (2023). Effect of progressive resistance exercise using Thera-band on edema volume, upper limb function, and quality of life in patients with breast cancer-related lymphedema. J. Exerc. Rehabil..

[14] Bock K., Ludwig R., Vaduvathiriyan P., Kluding P., Siengsukon C. (2022). Sleep disturbance in cancer survivors with lymphedema: A scoping review. Support. Care Cancer.

[15] Bock K., Peltzer J., Liu W., Colgrove Y., Smirnova I., Siengsukon C. (2025). Sleep quality and lymphedema in breast cancer survivors: A mixed method analysis. J. Cancer Surviv..

[16] Soltanipur M., Yarmohammadi H., Abbasvandi F., Montazeri A., Sheikhi Z. (2024). Sleep quality and risk of obstructive sleep apnea among breast cancer survivors with and without lymphedema. Sleep Breath..

[17] Keskin Kavak S., Kavak E.E. (2024). Fatigue and sleep quality improvement through complete decongestive therapy in postmastectomy lymphedema: An investigative analysis. Support. Care Cancer.

[18] Arslan E., Şevgin Ö. (2024). The effects of aerobic and oropharyngeal exercises on sleep quality of patients with obstructive sleep apnoea syndrome: A randomized controlled study. Sleep Breath..

[19] Buğday B., Çelik A.L., Safran E.E., Şevgin Ö. (2025). Impact of resistance exercise and diet on physical activity, sleep, and fatigue in obese individuals: A randomized controlled trial. BMC Public Health.

[20] Schulz K.F., Altman D.G., Moher D., the CONSORT Group (2010). CONSORT 2010 statement: Updated guidelines for reporting parallel group randomised trials. BMJ.

[21] Thomas M., Müller T., Busse M.W. (2005). Quantification of tension in Thera-Band® and Cando® tubing at different strains and starting lengths. J. Sports Med. Phys. Fit..

[22] Campbell K.L., Winters-Stone K.M., Wiskemann J., May A.M., Schwartz A.L., Courneya K.S., Zucker D.S., Matthews C.E., Ligibel J.A., Gerber L.H. (2019). Exercise guidelines for cancer survivors: Consensus statement from international multidisciplinary roundtable. Med. Sci. Sports Exerc..

[23] Deltombe T., Jamart J., Recloux S., Legrand C., Vandenbroeck N., Theys S., Hanson P. (2007). Reliability and limits of agreement of circumferential, water displacement, and optoelectronic volumetry in the measurement of upper limb lymphedema. Lymphology.

[24] Düger T., Yakut E., Öksüz Ç., Yörükan S., Bilgütay B.S., Ayhan Ç., Leblebicioğlu G., Kayıhan H., Kırdı N., Yakut Y. (2006). Kol, Omuz ve El Sorunları (Disabilities of the Arm, Shoulder and Hand—DASH) Anketi Türkçe uyarlamasının güvenirliği ve geçerliği. Fiz. Rehabil..

[25] Keeley V., Crooks S., Locke J., Veigas D., Riches K., Hilliam R. (2010). A quality of life measure for limb lymphoedema (LYMQOL). J. Lymphoedema.

[26] Bakar Y., Tuğral A., Özdemir Ö.Ç., Duygu E., Üyetürk Ü. (2017). Translation and validation of the Turkish version of Lymphedema Quality of Life Tool (LYMQOL) in patients with breast cancer related lymphedema. Eur. J. Breast Health.

[27] Buysse D.J., Reynolds C.F., Monk T.H., Berman S.R., Kupfer D.J. (1989). The Pittsburgh Sleep Quality Index: A new instrument for psychiatric practice and research. Psychiatry Res..

[28] Ağargün M.Y., Kara H., Anlar Ö. (1996). Pittsburgh Uyku Kalitesi İndeksi’nin geçerliği ve güvenirliği. Türk Psikiyatri Derg..

[29] Son W.C., Kim S.A., Kim A.H., Cheon H., Jeon J.Y. (2024). Effects of forearm resistance exercises on breast cancer-related lymphedema using segmental bioelectrical impedance analysis: A pilot randomized controlled trial. J. Clin. Med..

[30] Yin H., Wu W., Zhang Q., Xie F. (2025). Effect of home exercise on prevention and treatment of lymphedema in breast cancer patients. Front. Oncol..

[31] Luan B., Li Z., Yang Q., Xu Z., Chen Y., Wang M., Chen W., Ge F. (2024). The effects of ACSM-based exercise on breast cancer-related lymphoedema: A systematic review and meta-analysis. Front. Physiol..

[32] Stanton A.W.B., Modi S., Thomas M., Britton T.M.B., Purushotham A.D., Peters A.M., Levick J.R., Mortimer P.S. (2009). Lymphatic drainage in the muscle and subcutis of the arm after breast cancer treatment. Breast Cancer Res. Treat..

[33] Hahn B.A., Kleeven A., Richir M.C., Witkamp A.J., Kuijpers A.M.J., de Jong T., Qiu S., Coert J.H., Krijgh D.D. (2025). Objectifying clinical outcomes after lymphaticovenous anastomosis and vascularized lymph node transfer in the treatment of extremity lymphedema: A systematic review and meta-analysis. Microsurgery.

[34] Kappos E.A., Fabi A., Halbeisen F.S., Abu-Ghazaleh A., Stoffel J., Aufmesser-Freyhardt B., Bukowiecki J., Handschin T.M., Andree C., Haug M.D. (2025). Vascularized lymph node transfer (VLNT) versus lymphaticovenous anastomosis (LVA) for chronic breast cancer-related lymphedema (BCRL): A retrospective cohort study of effectiveness over time. Breast Cancer Res. Treat..

