# Supplementary material for: Effectiveness of Home-Based Progressive Resistance Exercise in the Management of Breast Cancer-Related Lymphedema: A Randomized Controlled Trial
Source: J Clin Med. 2026 Sep 13;15(18):7109. doi: 10.3390/jcm15187109 (PMC13607627; doi:10.3390/jcm15187109)
Supplement: Supplementary file 1 [file jcm-15-07109-s001.zip › jcm-4523053-supplementary.pdf]

Table S1. Individual baseline demographic and clinical characteristics of the study participants (*n* = 50).

ID	Group	Age (years)	Height (cm)	Body mass (kg)	BMI (kg/m²)	No. of children	Time since surgery (months)	Affected limb	Occupation	Education	Income	Marital status	Comorbidities	Regular medication use	History of psychological disorder	Baseline sleep quality (PSQI class)
E01	Exercise	57	152	61	26.4	3	12	Left	Nurse	High school	Low	Married	Hypertension	No	No	Poor
E02	Exercise	40	173	61	20.4	1	8	Right	Homemaker	Primary school	Low	Single	None	No	No	Poor
E03	Exercise	39	161	61	23.5	1	27	Right	Child development specialist	University degree	Middle	Widowed	Hypothyroidism	Yes	Yes	Good
E04	Exercise	38	152	64	27.7	2	32	Right	Nurse	University degree	Middle	Married	None	No	No	Good
E05	Exercise	47	153	67	28.6	1	14	Left	Retired	University degree	Middle	Married	Hypothyroidism	Yes	No	Poor
E06	Exercise	54	169	68	23.8	3	32	Left	Cleaning staff	High school	Low	Married	None	No	No	Poor
E07	Exercise	44	151	89	39.0	2	8	Left	Tailor	High school	Middle	Married	None	No	No	Good
E08	Exercise	49	174	72	23.8	1	46	Left	Not employed	Associate degree	High	Married	None	No	No	Good
E09	Exercise	57	171	64	21.9	1	47	Left	Textile worker	High school	High	Married	Type 2 diabetes	Yes	No	Good
E10	Exercise	51	152	90	39.0	2	25	Right	Accounting specialist	Associate degree	Middle	Widowed	Hypothyroidism	Yes	No	Poor
E11	Exercise	49	168	61	21.6	2	25	Right	Cashier	University degree	Middle	Married	Hypothyroidism	Yes	No	Poor
E12	Exercise	37	152	60	26.0	1	32	Left	Retired	Middle school	Middle	Married	Hypertension	Yes	Yes	Poor
E13	Exercise	50	157	86	34.9	1	15	Left	Homemaker	High school	Middle	Married	None	No	No	Poor
E14	Exercise	54	170	56	19.4	1	13	Right	Homemaker	High school	Low	Single	Hypertension	Yes	No	Good
E15	Exercise	42	169	65	22.8	3	18	Right	Call center worker	High school	Low	Married	Hyperlipidemia	No	No	Poor
E16	Exercise	38	170	60	20.8	4	46	Left	Retired	Primary school	High	Married	None	No	No	Poor
E17	Exercise	37	155	91	37.9	2	12	Right	Self-employed/tradesperson	University degree	High	Divorced	None	No	Yes	Poor
E18	Exercise	60	171	59	20.2	2	22	Right	Cleaning staff	Primary school	Middle	Married	Hyperlipidemia	Yes	No	Poor
E19	Exercise	59	159	66	26.1	0	10	Right	Retired	University degree	Middle	Single	Hypertension	Yes	No	Good
E20	Exercise	46	154	92	38.8	3	44	Right	Retired	Primary school	Middle	Married	Hypertension	Yes	No	Poor
E21	Exercise	45	165	71	26.1	2	18	Right	Teacher	University degree	Middle	Married	None	No	No	Good
E22	Exercise	58	159	74	29.3	3	28	Left	Retired	High school	Middle	Married	Hypertension	Yes	No	Poor
E23	Exercise	47	168	67	23.7	1	20	Right	Office staff	Associate degree	Middle	Divorced	None	No	No	Poor
E24	Exercise	52	161	80	30.9	2	36	Left	Homemaker	High school	Low	Married	Type 2 diabetes	Yes	No	Good
E25	Exercise	41	170	64	22.1	0	10	Right	Nurse	University degree	High	Single	Hypothyroidism	Yes	No	Poor
C01	Control	52	169	84	29.4	3	10	Right	Retired	University degree	Low	Married	Hyperlipidemia	Yes	No	Poor
C02	Control	42	172	63	21.3	2	6	Right	Human resources specialist	University degree	High	Widowed	None	No	No	Good
C03	Control	40	157	73	29.6	2	21	Right	Not employed	University degree	Middle	Married	None	No	Yes	Poor
C04	Control	47	155	79	32.9	0	13	Left	Bank employee	University degree	Low	Single	Asthma	Yes	No	Good
C05	Control	58	165	73	26.8	3	28	Right	Homemaker	Associate degree	Middle	Widowed	Hypertension	Yes	No	Poor
C06	Control	56	153	67	28.6	2	27	Right	Not employed	Postgraduate	Middle	Married	Hypothyroidism	Yes	No	Poor
C07	Control	47	173	75	25.1	4	27	Right	Child development specialist	University degree	Middle	Married	Hypothyroidism	Yes	No	Poor
C08	Control	58	152	62	26.8	0	33	Left	Retired	High school	Middle	Single	None	No	No	Poor
C09	Control	54	170	77	26.6	2	14	Left	Laboratory technician	University degree	Middle	Married	Hypertension	Yes	No	Poor
C10	Control	38	159	72	28.5	1	32	Left	Pharmacy technician	University degree	Low	Single	Migraine	Yes	No	Good

ID	Group	Age (years)	Height (cm)	Body mass (kg)	BMI (kg/m²)	No. of children	Time since surgery (months)	Affected limb	Occupation	Education	Income	Marital status	Comorbidities	Regular medication use	History of psychological disorder	Baseline sleep quality (PSQI class)
C11	Control	54	151	88	38.6	2	47	Left	Secretary	High school	Middle	Married	Hyperlipidemia	Yes	No	Poor
C12	Control	55	157	57	23.1	2	21	Right	Homemaker	Associate degree	Middle	Married	Hypothyroidism	Yes	Yes	Poor
C13	Control	46	163	67	25.2	3	29	Right	Homemaker	Middle school	High	Married	Hypertension	No	No	Poor
C14	Control	36	154	55	23.2	0	11	Left	Homemaker	Middle school	High	Married	Migraine	Yes	No	Good
C15	Control	45	168	63	22.3	1	39	Left	Self-employed/tradesperson	High school	High	Married	Hypothyroidism	Yes	Yes	Good
C16	Control	50	160	90	35.2	1	34	Left	Sales consultant	High school	Low	Divorced	Hypothyroidism	Yes	No	Good
C17	Control	58	161	71	27.4	2	38	Right	Tailor	High school	Middle	Married	Hypertension	Yes	No	Poor
C18	Control	46	166	65	23.6	2	12	Left	Homemaker	High school	Low	Married	None	No	No	Poor
C19	Control	44	163	88	33.1	1	10	Left	Engineer	University degree	Middle	Single	None	No	No	Good
C20	Control	40	167	92	33.0	2	46	Left	Homemaker	Middle school	Low	Married	None	No	Yes	Poor
C21	Control	44	164	69	25.7	1	17	Left	Secretary	High school	Middle	Married	None	No	No	Poor
C22	Control	60	158	76	30.4	3	30	Right	Retired	Middle school	Low	Married	Hypertension	Yes	No	Poor
C23	Control	48	167	66	23.7	2	22	Left	Accounting specialist	University degree	Middle	Married	None	No	No	Poor
C24	Control	55	160	82	32.0	2	34	Right	Homemaker	High school	Low	Widowed	Type 2 diabetes	Yes	No	Poor
C25	Control	42	171	65	22.2	0	12	Left	Bank employee	University degree	High	Single	Hypothyroidism	Yes	No	Poor

E01–E25, exercise group; C01–C25, control group; BMI, body mass index (calculated from height and body mass, rounded to one decimal place); PSQI, Pittsburgh Sleep Quality Index (poor sleep quality defined as PSQI global score > 5). Summary statistics in Table 1 of the main text are means ± sample standard deviations (n − 1 denominator) of the values listed here.
